# MicroRNA Detection by DNA‐Mediated Liposome Fusion

**DOI:** 10.1002/cbic.201700592

**Published:** 2018-01-15

**Authors:** Coline Jumeaux, Olov Wahlsten, Stephan Block, Eunjung Kim, Rona Chandrawati, Philip D. Howes, Fredrik Höök, Molly M. Stevens

**Affiliations:** ^1^ Department of Materials Department of Bioengineering, and Institute of Biomedical Engineering Imperial College London Exhibition Road London SW7 2AZ UK; ^2^ Department of Physics Chalmers University of Technology 41296 Göteborg Sweden; ^3^ Present address: School of Chemical and Biomolecular Engineering The University of Sydney Sydney NSW 2006 Australia; ^4^ Present address: Department of Chemistry and Biochemistry Freie Universität Berlin 14195 Berlin Germany

**Keywords:** biosensors, FRET, liposomes, membrane fusion, nucleic acids

## Abstract

Membrane fusion is a process of fundamental importance in biological systems that involves highly selective recognition mechanisms for the trafficking of molecular and ionic cargos. Mimicking natural membrane fusion mechanisms for the purpose of biosensor development holds great potential for amplified detection because relatively few highly discriminating targets lead to fusion and an accompanied engagement of a large payload of signal‐generating molecules. In this work, sequence‐specific DNA‐mediated liposome fusion is used for the highly selective detection of microRNA. The detection of miR‐29a, a known flu biomarker, is demonstrated down to 18 nm within 30 min with high specificity by using a standard laboratory microplate reader. Furthermore, one order of magnitude improvement in the limit of detection is demonstrated by using a novel imaging technique combined with an intensity fluctuation analysis, which is coined two‐color fluorescence correlation microscopy.

The detection of target molecules in a specific and sensitive manner is of critical importance for the development of efficient disease diagnostic devices. Nature is a great source of inspiration for the design of such platforms because throughout the course of evolution highly sensitive and specific sensing or signaling processes have emerged that use refined components made of only a few molecular building blocks.^1^ One of these mechanisms is the fusion of lipid bilayers; an essential process that allows the transfer of chemicals through an otherwise impervious barrier,^2^, ^3^ which facilitates inter‐ and intracellular communication.^4^ In the case of neuronal fusion, in which Ca^2+^‐triggered neurotransmitter release into the synaptic cleft from a vesicle occurs on the sub‐millisecond timescale,^5^ complex molecular machinery facilitates this highly regulated process, and proteins belonging to the SNARE family (soluble *N*‐ethylmaleimide‐sensitive factor attachment protein receptors) have emerged as the key components to facilitate the molecular recognition and fusion of bilayers.^6^, ^7^, ^8^

An increased understanding of biological processes and the design principles underlying recognition enables the progression of the field of the design of biomimetic materials, which harness the power and efficiency of natural processes, combined with biological building blocks (e.g., DNA, RNA, peptides, proteins, lipids) to create hierarchically organized materials, and the development of a new generation of sensors.^1^

Membrane fusion has been extensively studied by using model systems, such as proteoliposomes, in which native fusogenic proteins are exposed on the surface of artificial lipid vesicles (liposomes).^9^, ^10^, ^11^, ^12^, ^13^, ^14^ Another approach involves the design of biologically inspired constructs to trigger liposome docking and membrane fusion. Simplified synthetic analogues are an excellent tool to increase the understanding of the fusion process at the atomistic level, and each segment of the synthetic construct can be varied to study its role in the occurrence of fusion.^2^ Synthetic analogues enabling specific molecular recognition used to trigger membrane fusion include coiled‐coil forming peptides^3^, ^15^ and DNA.^16^, ^17^, ^18^, ^19^ Cholesterol‐terminated double‐stranded DNA (dsDNA) has been shown to facilitate efficient self‐insertion into liposomes,^20^ and that their hybridization, if designed to occur in a zipper‐like fashion, can induce liposome–liposome fusion (Figure S1 in the Supporting Information).^18^, ^19^ DNA, thanks to its high selectivity in sequence‐guided self‐assembly, has great potential for biosensing applications. Attractive properties of liposomes, such as ease of functionalization and biocompatibility, endow them with promising applications in biomedical and biotechnology fields.^21^, ^22^, ^23^ Furthermore, with relatively few targets leading to fusion and engagement of a large payload of signaling molecules (e.g., FRET‐active dyes), it is reasonable to assume that target‐mediated liposome fusion could lead to highly amplified detection. Inspired by this reasoning, we have engineered a sensing platform for detecting a clinically relevant biomarker for influenza virus infection,^24^ microRNA‐29a (miR‐29a), in a highly specific and sensitive manner by DNA‐mediated liposome membrane fusion. This work, conjointly with a recent report demonstrating the possibility of triggering peptide nucleic acid mediated liposome fusion with oligonucleotides,^25^ constitutes the first example of biomarker‐triggered liposome fusion.

Herein, we encapsulated FRET donor–acceptor pairs, DiI and DiD (Figure S2), in the membrane of DNA‐functionalized liposomes. In this assay, liposomes containing either DiI or DiD FRET pairs in the membrane are functionalized with self‐inserting, cholesterol‐terminated dsDNA (Figure 1). A hairpin DNA (H, green strand in Figure 1) is designed to block the sticky end of dsDNA A/B (ds‐A/B), which prevents hybridization with dsDNA C/D (ds‐C/D) and inhibits liposome docking and fusion. The hairpin DNA is also specially designed to include a region containing a complementary sequence with miR‐29a. Hence, in the presence of target miRNA, hybridization with hairpin DNA reveals the sticky end of ds‐A/B, which can then hybridize with ds‐C/D to initialize liposome docking and membrane fusion. This is then detected by an increase in FRET signal. The reported liposome fusion mechanism for oligonucleotide detection presents several key advantages in its experimental design. It is a homogeneous assay, operated at room temperature, and involving relatively few experimental steps. Notably, other liposome‐based assays for oligonucleotide detection require the destruction of liposomes through the addition of membrane‐disrupting agents.^21^, ^26^ Herein, similarly to assays developed by Jakobsen et al.,^27^, ^28^ the readout is obtained without the need for additional separation, amplification, and washing steps, which has great potential for further developing this assay towards point‐of‐care applications.

**Figure 1:**
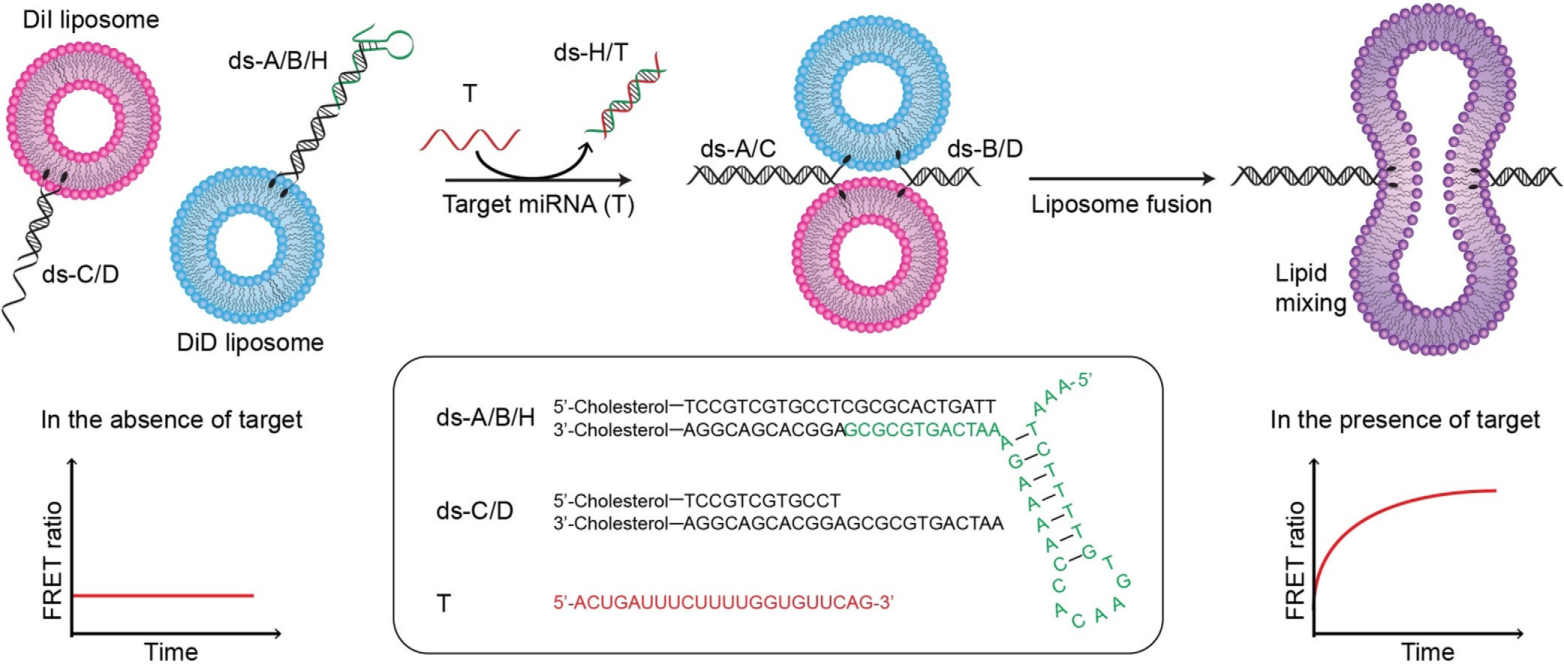
Schematic illustration for the process of miRNA detection based on the promotion of DNA‐mediated liposome fusion. Target miRNA (red strand) is complementary to the hairpin (green strand) and hybridizes to it; this displacement reveals the sticky end of ds‐A/B. In the presence of target miRNA, liposome fusion is promoted by ds‐A/B and ds‐C/D hybridizing in a zipper‐like manner, which results in an increase in FRET signal. In the absence of target miRNA, liposome fusion is inhibited because hairpin DNA remains hybridized on ds‐A/B and no FRET increase is observed.

In this assay, schematically illustrated in Figure 1, the hairpin DNA strand (H) is designed to hybridize on the sticky end of a duplex and to be displaced in the presence of target miRNA. This novel mechanism of a dual‐function hairpin DNA is an advantageous strategy because it offers a unique site for target hybridization, which is independent of the zippering regions; therefore, its design is universal and can be tailored to adapt to a broad spectrum of target biomarkers. It is crucial to optimize the design of H to meet the following three requirements: 1) unbound H must maintain its structural stem‐loop feature (not a random coil structure), and have at least one arm of the stem that is part of the recognition sequences; 2) H must be strongly hybridized to ds‐A/B in the absence of target, which prevents the hybridization of A with C and further unzipping of the dsDNAs; and 3) H must be displaced only by the specific target miRNA (T), revealing the sticky end of ds‐A/B, which will initiate liposome docking by hybridization with ds‐C/D.

We designed three hairpin structures: H_6_, H_7_, and H_13_ (sequences in Table S1). The optimization of the hairpin design is summarized in section S5 (Supporting Information). Briefly, first, we visualized the predicted secondary structures formed by the hybridization of H, A, and B (Figure S3) by using algorithms provided in the NUPACK software,^29^ which also showed that out of the three predicted constructs, ds‐A/B/H_7_ had the highest probability of formation (Table S2). Furthermore, the hybridization and displacement of H in solution were characterized by means of native PAGE analysis (Figure S4). We demonstrated that only H_7_ showed both efficient hybridization with ds‐A/B and displacement by T; therefore, H_7_ was chosen as the hairpin structure for the liposome fusion assay.

Following selection of H_7_, we translated the hairpin strand displacement mechanism in the presence of target miR‐29a into a liposome fusion assay. The majority of methods for detecting membrane fusion are based on fluorescence generation or FRET, and careful choice of the reporter assay allows selective discrimination between lipid mixing (inner or outer lipid leaflet) and content mixing.^30^ To maximize signal generation during the assay, we selected a lipid‐mixing assay to report fusion events because studies have shown that a high level of lipid mixing can occur with a limited degree of content mixing.^17^ DiI and DiD FRET pairs are based on Cy3 and Cy5 cyanine dyes, respectively (Figure S2), and contain long alkyl chains that render them highly lipophilic; therefore, they will be included in the membrane of liposomes. Two populations of liposomes containing either DiI or DiD were mixed; upon membrane fusion, these fluorescent dyes mixed, resulting in FRET signal generation.

DiD and DiI liposomes were functionalized with ds‐A/B/H_7_ and ds‐C/D, respectively, and we studied the ability of miR‐29a to displace H_7_ and trigger liposome fusion (Figure 2). DiD liposomes were incubated with various quantities of miR‐29a. Then, equal volumes of DiI and DiD liposomes were mixed and the evolution of the FRET ratio was measured over time (Figures 2 A and S5). In the absence of miR‐29a, the FRET ratio slightly increased during the first 20 min of measurement then plateaued, which showed that H_7_ was successfully hybridized on ds‐A/B and was able to prevent liposome fusion. As the quantity of miR‐29a increased, the FRET ratio and its gradient in the first 20 min increased; this demonstrated that miR‐29a was able to displace H_7_ and trigger liposome fusion. To study the sensitivity of the assay, we plotted the dose–response curve obtained after 30 min of incubating DiI and DiD liposomes (Figure 2 B). The limit of detection (LOD) was calculated by first determining the *z* value (*z*=blank+3*σ*), in which *σ* is the standard deviation of the blank. The LOD was determined to be 18 nm by reporting the *z* value in the equation of the curve fitted to the dose–response measurements.

**Figure 2:**
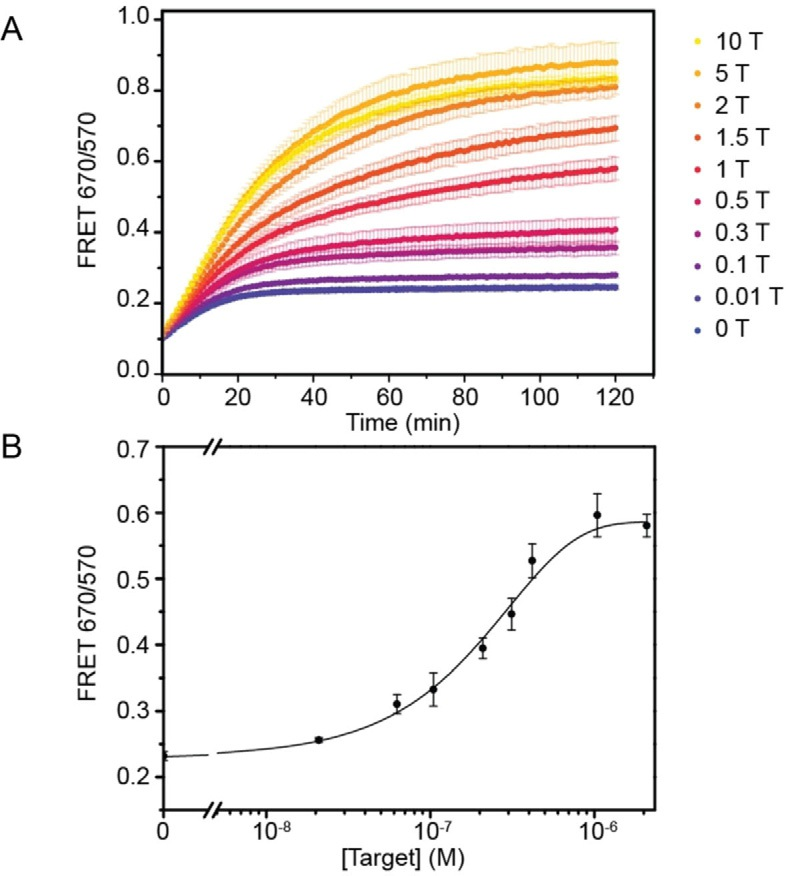
Evaluation of the sensitivity of the DNA‐mediated liposome fusion assay for miR‐29a detection: A) Evolution of the FRET signals over time showing the kinetics of DNA‐mediated liposome fusion in the presence of different concentrations of target miR‐29a (1 T corresponds to 2.1×10^−7^ m), and B) corresponding dose–response curve obtained after 30 min of mixing DiI‐ and DiD‐labeled DNA‐functionalized liposomes (*n*=3). Error bars represent standard deviation.

Next, the specificity of the assay was studied (Figure 3). The miR‐29 family members are important regulators of human diseases,^31^ and include miR‐29a, miR‐29b‐1, miR‐29b‐2, and miR‐29c. In a microarray assay, the expression levels of both miR‐29a and miR‐29b were significantly different between critically ill patients with H1N1 infection and healthy controls.^24^ We tested the ability of the assay to discriminate between the fully complementary target, miR‐29a, and its miR‐29 family counterparts, miR‐29b and miR‐29c. The sequences of miR‐29b and miR‐29c differ from that of miR‐29a by several mismatching and additional nucleotides (Table S1). Therefore, to study the specificity of our assay, we designed artificial sequences, differing from miR‐29a by 1, 2, or 3 nucleotides: miR‐29a‐1MM, miR‐29a‐2MM, and miR‐29a‐3MM (Table S1). DiD liposomes functionalized with ds‐A/B/H_7_ were incubated with one molar equivalent of different miRNAs. Subsequently, equal volumes of DiI and DiD liposomes were mixed, and the evolution of the FRET signal was measured over time (Figures 3 A and S6). Only the fully complementary target, miR‐29a, was able to trigger liposome fusion, whereas no other sequences showed a significant increase in the FRET ratio, relative to that of the signal measured in the absence of target. Figure 3 B summarizes the relative values of the FRET ratios at 30 min of incubation obtained with each miRNA, relative to the control, and showed that our assay had a high specificity because we were able to discriminate between sequences with one nucleotide mismatch.

**Figure 3:**
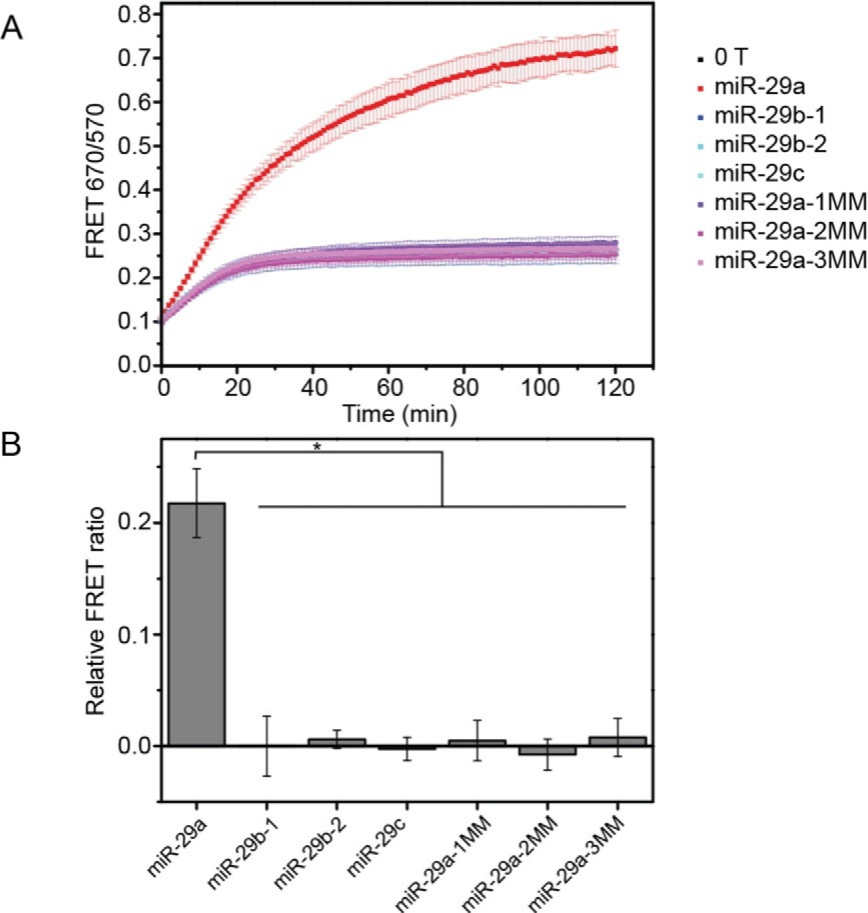
Evaluation of the specificity of the DNA‐mediated liposome fusion assay for miR‐29a detection: A) Evolution of the FRET signals over time showing the kinetics of DNA‐mediated liposome fusion in the presence of various miRNA sequences at a concentration of 2.1×10^−7^ m. B) Corresponding bar chart representing the FRET ratio values relative to the control for each miRNA sequence 30 min after mixing DiI‐ and DiD‐labeled DNA‐functionalized liposomes. *n*=3, * *p*<0.05 (ANOVA followed by Tukey post‐hoc test). Error bars represent standard deviation.

This high specificity is attributed to the fact that displacing the hairpin requires high stringency.^32^ Although the LOD value reported herein is much higher than that obtained with other miRNA detection methods based on enzymatic target amplification,^33^, ^34^ this assay remarkably distinguished strong sequence homologies, which is typically difficult to achieve for homogeneous assays that do not incorporate washing, amplification, or separation steps.^35^ Additionally, the DNA‐mediated liposome fusion assay reduces the complexity and risk of errors that would otherwise be associated with enzymatic target amplification.^36^

To further the understanding of the assay for miRNA detection and potentially gain in detection limit and/or minimize material consumption, a suspension‐based two‐color fluorescence correlation microscopy method was developed that allowed extraction of FRET signals from imaging low numbers of liposomes (<50).^37^ The experimental setup is presented in section S7 (Supporting Information). In brief, it comprises a regular fluorescence microscope equipped with a beam splitter (Figure S7 a), which enables the simultaneous separation of the emitted light originating from DiI liposomes (centered around *λ*=570 nm; denoted as the “green channel” in the following) and fused DiI and DiD liposome complexes (centered around *λ*=670 nm; “red channel”) upon direct excitation of DiI liposomes by a laser source (*λ*=488 nm).

We exploited the increased sensitivity in terms of liposome concentration of the two‐color fluorescence microscopy setup and mixed liposome solutions at 10 pm concentration for incubation with the target, and then diluted the solutions to 1 pm before performing the measurements (compared with 1.7 nm liposome concentration used for the microplate reader setup).

Due to its large conceptual similarity to that of fluorescence correlation spectroscopy (FCS),^38^, ^39^, ^40^ we tried to assess FRET characteristics from the intensity fluctuations of *I*_g_(*t*) and *I*_r_(*t*) (intensity traces integrated over the entire green and red channels, respectively). In FCS, the average number, *N*, of fluorescent particles within the readout volume (usually a diffraction‐limited spot due to the usage of a confocal excitation scheme) can be obtained by calculating the autocorrelation function, *g*, of the recorded fluorescence intensity trace, *I*(*t*) [Eq. (1)],

(1) $$g\left(\tau \right)=\frac{\left\langle I(t)\cdot I(t+\tau )\right\rangle }{\left\langle I\left(t\right)\right\rangle {}^{2}}-1$$

and by employing *N*=1/*g*(0).^41^ As shown in section S7 (Supporting Information), the same methodology can be applied to the intensity traces obtained from our microscope setup, if the autocorrelation function of the intensity ratio *I*_r_(*t*)/*I*_g_(*t*) is calculated. This normalization was necessary because fluctuations in *I*_r_(*t*) were caused by two processes, fused FRETing liposomes and bleed‐through of DiI liposomes, making *g*(0) more sensitive to the number of DiI liposomes than that of fused FRETing DiD liposomes (section S7). Because DiI liposomes cause correlated fluctuations in *I*_g_(*t*) and *I*_r_(*t*), the use of *I*_r_(*t*)/*I*_g_(*t*) eliminates the sensitivity of DiI liposomes, but boosts FRET‐based fluctuations (Figure 4 A), making *g*(0) mainly sensitive to the number of fused FRETing DiD liposomes (section S7 and Figure S8). Representative examples for the autocorrelation function of *I*_r_(*t*)/*I*_g_(*t*) are shown in Figure 4 B, clearly indicating a decrease in *g*(0) with increasing miR‐29a concentration, which is, due to *N≈*1/*g*(0), indicative of an increase in the number of fused FRETing DiD liposomes, *N*_FRET_, in the field of view. Calibration of this approach with solutions containing only FRETing vesicles of known bulk concentration, *c*_FRET_ (Figure S9), allowed us to extract a dose–response curve (Figure 4 C) similar to that of the microplate reader assay. The LOD obtained with the two‐color fluorescence correlation microscopy setup was determined to be 1.2 nm, which was a one order of magnitude improvement in sensitivity compared with that of ensemble measurements obtained by using the microplate reader setup. Hence, two‐color fluorescence correlation microscopy has in its current implementation an improved performance relative to that of the microplate reader assay (in terms of LOD), and consumes three orders of magnitude less material thanks to its ability to operate at liposome concentrations as low as 1 pm (in contrast to 1.7 nm used in the microplate reader assay).

**Figure 4:**
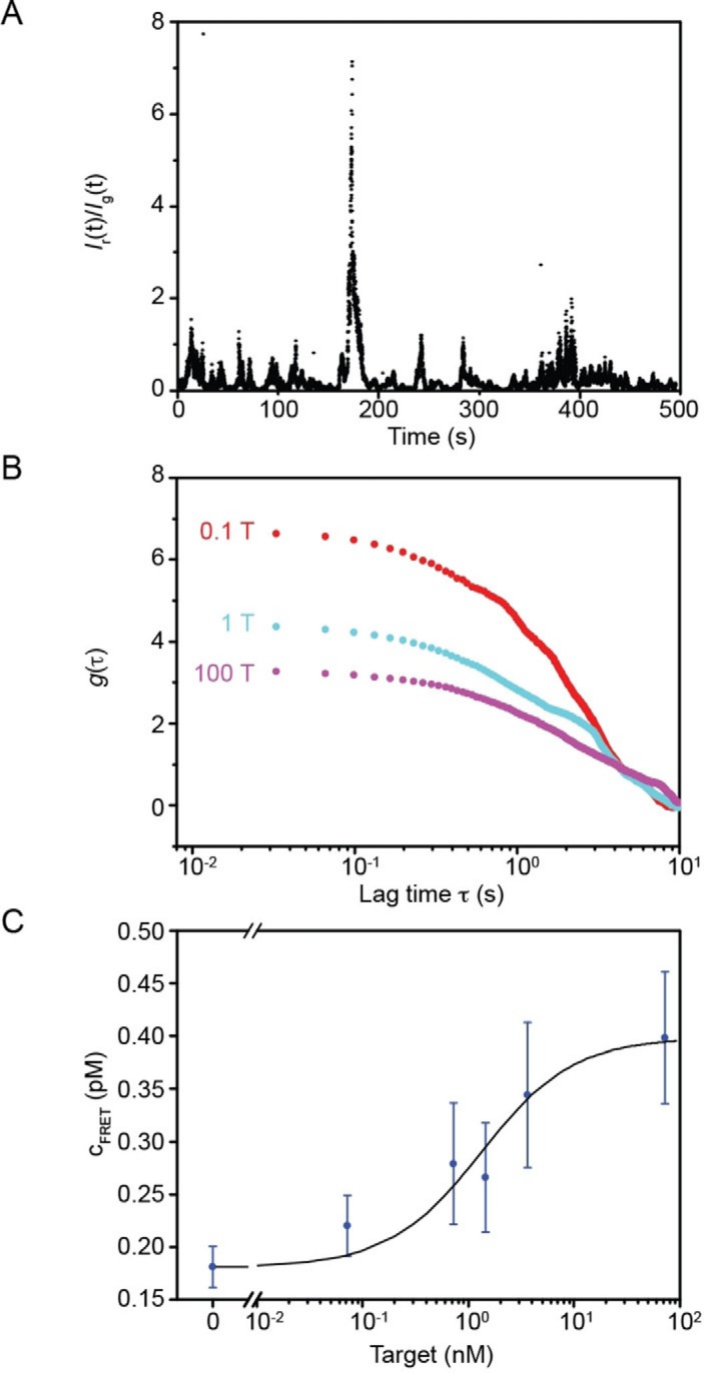
A) The *I*_r_(*t*)/*I*_g_(*t*) ratio of the total intensity of the red channel over the green channel. B) Representative autocorrelation functions of *I*_r_(*t*)/*I*_g_(*t*)−*γ* (with *γ*=0.065 as the average bleed‐through factor), for miR‐29a concentrations indicated (1 T corresponds to 7.2×10^−10^ m). C) Dose–response curve obtained by using the two‐color fluorescence correlation microscopy setup (averaged over three independent sample sets, covering at least five measurements each). Error bars represent SEM. See Section S7 in the Supporting Information for details of the entire process.

In summary, we set out to explore whether DNA‐mediated liposome fusion could be controlled by interactions with specific nucleic acid targets, and whether this mechanism could form the basis of a detection assay. We successfully demonstrated sensitive and specific detection of miR‐29a at nanomolar concentrations by using a FRET‐based fluorescence output, and explored two methods of analyzing the fluorescence signal. With a standard laboratory microplate reader, a LOD of 18 nm was obtained, whereas, with two‐color fluorescence correlation microscopy combined with an intensity fluctuation analysis, we observed a LOD improved by one order of magnitude. Liposome and analyte concentrations that were three orders of magnitude lower than those used in the microplate reader assay were employed, and demonstrated the utility of such a setup in both the study of liposome fusion and application in a biosensing system. Furthermore, our system has potential in the development of new diagnostic platforms targeting miRNA and other nucleic acids. By optimizing the highly promising concept of relatively few targets leading to fusion and activation of a large payload of signal‐generating molecules, the LOD could be much improved in subsequent iterations. Factors such as assay temperature, dsDNA coverage, and choice of reporter signal are expected to improve the sensitivity in our assay, and will be the subject of further research. Also, by tuning the design of the DNA hairpin, detection of diverse molecular targets with high specificity, combined with a simple experimental workflow, is possible.

## Conflict of interest

*The authors declare no conflict of interest*.

## Supporting information

As a service to our authors and readers, this journal provides supporting information supplied by the authors. Such materials are peer reviewed and may be re‐organized for online delivery, but are not copy‐edited or typeset. Technical support issues arising from supporting information (other than missing files) should be addressed to the authors.

Supplementary
