# Supplementary material for: MicroRNA Detection by DNA‐Mediated Liposome Fusion
Source: Chembiochem. 2018 Jan 15;19(5):434–8. doi: 10.1002/cbic.201700592 (PMC5861668; doi:10.1002/cbic.201700592)
Supplement: Supplementary file 1 — Supplementary [file CBIC-19-434-s001.pdf]

CHEMBIOCHEM

Supporting Information

MicroRNA Detection by DNA-Mediated Liposome Fusion

Coline Jumeaux,[a] Olov Wahlsten,[b] Stephan Block,[b, d] Eunjung Kim,[a] Rona Chandrawati,[a, c] Philip D. Howes,[a] Fredrik Höök,*[b] and Molly M. Stevens*[a]

cbic_201700592_sm_miscellaneous_information.pdf

Supporting Information
©Wiley-VCH 2016
69451 Weinheim, Germany

MicroRNA Detection by DNA-Mediated Liposome Fusion

Coline Jumeaux, Olov Wahlsten, Stephan Block, Eunjung Kim, Rona Chandrawati, Philip D. Howes, Fredrik Höök,* and Molly M. Stevens*

Abstract: Membrane fusion is a process of fundamental importance in biological systems, involving highly selective recognition mechanisms for trafficking of molecular and ionic cargos. Mimicking natural membrane fusion mechanisms for the purpose of biosensor development holds great potential for amplified detection, since relatively few highly discriminating targets lead to fusion and an accompanied engagement of a large payload of signal-generating molecules. In this work, we use sequence specific DNA-mediated liposome fusion for highly selective detection of microRNA. We demonstrate detection of miR-29a, a known flu biomarker, down to 18 nM within 30 minutes with high specificity using a standard laboratory microplate reader. Further, we demonstrate one order of magnitude improvement in the limit of detection by using a novel imaging technique combined with an intensity fluctuation analysis, coined 2-color fluorescence correlation microscopy.

DOI: 10.1002/anie.2016XXXXX

Table of Contents

S1. Experimental procedures 3
S2. Nucleic acid sequence 5
Table S1 5
S3. Mechanism of DNA-mediated liposome fusion 6
Figure S1 6
S4. Properties of the FRET pair used in this study 7
Figure S2 7
S5. Optimization of the hairpin sequence 8
Figure S3 8
Table S2 9
Figure S4 9
S6. Evaluation of the sensitivity and specificity for miR-29a detection 10
Figure S5 10
Figure S6 10
S7. Fluorescence correlation microscopy analysis 11
Figure S7 11
Figure S8 14
Figure S9 14
S8. Calculation of the LOD for the 2-color fluorescence microscopy setup 16
References 17

S1. Experimental procedures

Materials

1,2-Dioleoyl-*sn*-glycero-3-phosphocholine (DOPC) and 1,2-dioleoyl-*sn*-glycero-3-phosphoethanolamine (DOPE) lipids were purchased from Avanti Polar Lipids, Inc. Cholesterol, tris(hydroxymethyl)-aminomethane hydrochloride (Tris), sodium chloride (NaCl), ethylenediaminetetraacetic acid (EDTA), ammonium persulfate (APS), and tetramethylethylenediamine (TEMED) were obtained from Sigma-Aldrich. 1,1'-dioctadecyl-3,3,3',3'-tetramethylindodicar-bocyanine perchlorate, DiIC18(5) (DiD), 1,1-dioctadecyl-3,3,3',3'-tetramethylindocarbo-cyanine perchlorate, DiIC18(3) (DiI), Tris/boric acid/EDTA (TBE) buffer, 10 bp DNA ladder, BlueJuice gel loading buffer, and SYBR gold nucleic acid gel stain were purchased from Invitrogen Life Technologies. 30 % Acrylamide/bis solution (29:1) was obtained from Bio-Rad (USA). HPLC-purified oligonucleotides were purchased from IBA GmbH (Germany) and Integrated DNA Technologies (Belgium) (Supporting Table S1). Nuclease-free water (non DEPC-treated) was purchased from Thermo Fisher Scientific (AmbionTM).

Analysis of hairpin structures

Secondary structure analysis of hairpin DNA hybridized with dsDNA was performed by means of NUPACK analysis algorithms[1] using the Internet-based tool NUPACK nucleic acid package[2] (Figure S3).

Native polyacrylamide gel electrophoresis (PAGE) assay

To analyze hairpin hybridization with dsDNA and hairpin displacement by target miR-29a in solution, all the duplexes were formed in TE buffer containing 50 mM NaCl (pH 8.0) or TE buffer containing 150 mM NaCl (pH 7.4) for hairpin displacement, and resolved in 15 % native PAGE in 1× TBE buffer. For PAGE, 3 pmol of each mixture, combined with gel loading buffer, were loaded into each lane and the gels were run in 1× TBE buffer at 50 V for 10 min and 110 V for 65 min. The gels were post-stained with 1× SYBR gold for 30 min at room temperature and imaged using the BioSpectrum Imaging System (Ultra-Violet Products, Cambridge, UK).

Liposome preparation and functionalization

Liposomes were composed of DOPC/DOPE/Cholesterol (50:25:25 mass ratio) and contained 2 mol % of DiI or DiD. Dry lipid films (5 mg/mL) were hydrated in TE buffer (10 mM Tris, 1 mM EDTA, pH 8.0) containing 150 mM NaCl at 37 °C for 30 min. Liposomes were extruded using the Avanti Lipids Mini-Extruder with 100 nm polycarbonate membranes (Whatman). Each DNA strand (A and B, C and D) was mixed at 1:1 molar ratio in TE buffer (10 mM Tris, 1 mM EDTA, pH 7.9) containing 50 mM NaCl. To form ds-A/B, ds-C/D and H, the mixtures were annealed at 80 °C for 5 min, then allowed to hybridize by slowly cooling down the solution to 25 °C. Then ds-A/B and H were mixed at 1:1 molar ratio and left to hybridize at 25 °C for 1 h. DiD liposomes were functionalized with ds-A/B/H and DiI liposomes were functionalized with ds-C/D, both at a ratio of 100 dsDNA per liposome, by allowing the cholesterol modified dsDNA to self-incorporate into the liposomes for 1 h. Unbound DNAs were removed using Nanosep® Centrifugal Device with Omega Membrane (MWCO 30 kDa, 5 min x 5000 g, Pall).

Measurement of fusion kinetics

Liposomes were diluted to 1.7 nM in TE buffer containing 150 mM NaCl (pH 7.4). Various concentrations of target miR-29a (for evaluation of the sensitivity of the assay) or various other miRNAs (for evaluation of the specificity of the assay, see Supporting Table S1 for the detailed nucleic acids sequences) were added to solutions of DiD liposomes, and allowed to hybridize for 1 h at room temperature. For evaluation of the specificity, the concentration of miRNA was set to 2.1×10^{-7} M (1:1 molar equivalent). Then, equal volumes of DiI and DiD liposomes were mixed. Kinetics of

fusion of DiI and DiD liposomes were measured in 96-well plates, and fluorescence emission of the dyes was recorded with a SpectraMax M5 microplate reader (Molecular Devices, USA) at 25 °C, using λ_{ex} = 530 nm, $\lambda_{em,DiI}$ = 570 nm, $\lambda_{em,DiD}$ = 670 nm.

Measurement of the assay sensitivity using 2-color fluorescence microscopy setup

2-color fluorescence correlation microscopy analysis measurements were performed on an upright Olympus BX61 microscope equipped with a Hamamatsu ORCA-Flash4.0 V2 Digital CMOS camera (Figure S7). The sample containing the liposomes was introduced in a narrow sample channel, constructed by two thin glass slides separated by 2 mm using several layers of double-sided tape yielding a channel volume of ~100 µl. The sample was exposed to monochromatic light (488 nm) via a laser (max power: 150 mW, Cobolt) coupled to a single mode polarization maintaining optical fiber (P460-HP, Thorlabs) that was carefully inserted into the sample channel using a manual micrometer translational stage. The emitted light from the liposomes in solution was collected with an Olympus 20×, NA = 0.5, water immersion objective (UMPlanFL) and then passed through a dual emission image splitter (OptoSplit II, Cairn Research). The beam splitter was equipped with a filter cube containing a dichroic mirror (transmission > 650 nm, Cairn Research) and two fluorescence filters (590/50 nm and 700/75 nm, Chroma Technology Corporation). Additionally, a 10× objective (Olympus) was placed between the beam splitter and the CMOS camera. Images (4×4 binned) were acquired at 33 frames per second at 30 ms exposure time with a laser power of 50 mW.

Liposomes were diluted to 10 pM in TE buffer containing 150 mM NaCl (pH 7.4). DiI and DiD liposomes were mixed at equal volumes, and various concentrations of target miR-29a were added to the liposomes. These solutions were left to react for 1 h at room temperature, and then the liposomes were further diluted to 1 pM before measuring the amount of FRET in the 2-color fluorescence microscopy setup.

S2. Nucleic acid sequences

Table S1. Nucleic acid sequences used in this study.

Name	Sequence (5’ – 3’)	Modification	Supplier
A	TCCGTCGTGCCTCGCGCACTGATT	5’-Cholesterol	IBA GmbH
A’	TCCGTCGTGCCTATTGGGTGAATTATTGAGAG	5’-Cholesterol	IBA GmbH
B	AGGCACGACGGA	3’-Cholesterol	IBA GmbH
C	AATCAGTGCGCGAGGCACGACGGA	3’-Cholesterol	IBA GmbH
C’	CTCTCAATAATTCACCCAATAGGCACGACGGA	3’-Cholesterol	IBA GmbH
D	TCCGTCGTGCCT	5’-Cholesterol	IBA GmbH
H_6	*CTCTCAATAA***CACTGA**ACACCAAAAGAAA**TCAGTG***CACCCAAT*	-	IBA GmbH
H_{13}	*CTCTCAATAA***CTGAACACCAAAA**GAAATCAG**TTTTGGTGTTCAG***CACCCAAT*	-	IDT
H_7	AAA**TCTTTTG**CTGAACAC**CAAAAGA***AATCAGTGCGCG*	-	IDT
miR-29a	ACUGAUUUCUUUUGGUGUUCAG	-	IBA GmbH
miR-29b-1	GCUGGUUUCAUAUGGUGGUUUAGA	-	IDT
miR-29b-2	CUGGUUUCACAUGGUGGCUUAG	-	IDT
miR-29c	UGACCGAUUUCUCCUGGUGUUC	-	IDT
miR-29a-1MM	ACUGAUUUCUUCUGGUGUUCAG	-	IDT
miR-29a-2MM	ACUGAGUUCUUCUGGUGUUCAG	-	IDT
miR-29a-3MM	ACUGAGUUCUUCUGGUGCUCAG	-	IDT

Mismatches compared with the sequence of miR-29a are indicated in red.
Stem sequences are indicated in bold.
Nucleobases binding to the sticky end of A or A’ are indicated in italic.
Nucleobases binding to miR29-a are underlined.

S3. Mechanism of DNA-mediated liposome fusion

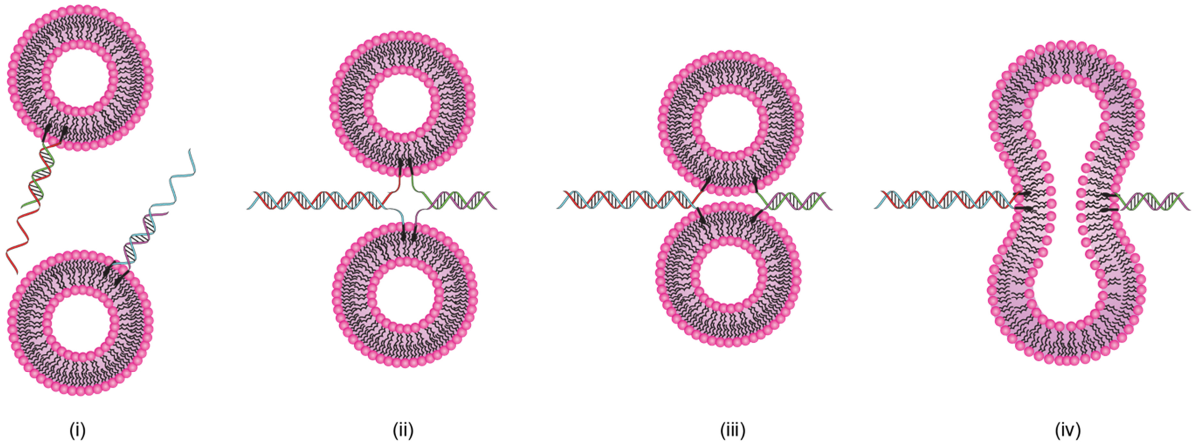

Figure S1. Mechanism of DNA-mediated liposome fusion. (i) Two separate populations of liposomes are functionalized with complementary DNA strands; (ii) and (iii) DNA hybridization in a zipper-like fashion brings two liposomes in close contact, eventually leading to mixing of outer leaflet lipids, and in some cases, (iv) opening of the fusion pore. Adapted from Stengel *et al.*[3]

S4. Properties of the FRET pair used in this study

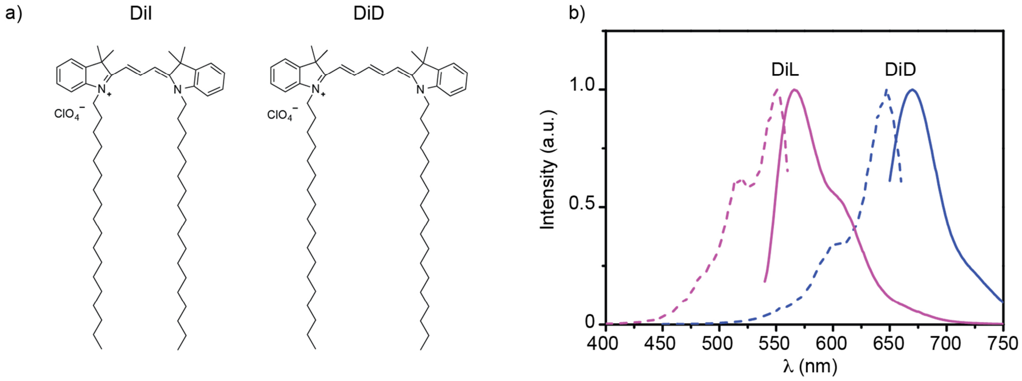

Figure S2. a) Chemical structures of DiI and DiD FRET pairs and b) absorbance (dashed lines) and fluorescence emission (full lines) spectra of DiI (λ_{ex} = 530 nm) and DiD (λ_{ex} = 647 nm).

S5. Optimization of the hairpin sequence

We designed three different hairpins: H_6, H_7, and H_{13}, and studied the influence of the length of the stem and the position of the hairpin stem on the sticky end of ds-A/B on the hybridization and displacement of the hairpin. H_6 and H_{13} have 6 and 13 base pairs in their stem, respectively, and are hybridized in a central position on the sticky end of ds-A/B, while H_7 has 7 base pairs in its stem and is hybridized in a terminal position on the sticky end of ds-A/B (sequences in Table S1). The sequence of the long single stranded DNA (ssDNA) in ds-A/B was identical in the case of H_6 and H_{13} and is referred as A'. For H_7 the sticky end of dsDNA was shortened to 12 bases in order to favor the displacement of the hairpin by miR-29a and the corresponding long ssDNA strand is referred to as A. We used algorithms provided in the NUPACK software[1] to visualize the predicted secondary structures formed by A or A' (long ssDNA), B (short ssDNA) and H (hairpin) (Figure 2a). Out of the three predicted constructs, ds-A/B/H_7 had the highest probability of formation followed by ds-A'/B/H_6 then ds-A'/B/H_{13} (Figure S3 and Table S2); the base-pairing probability at the base of the stem was lower when the hairpin hybridized in the centre of the dsDNA sticky end.

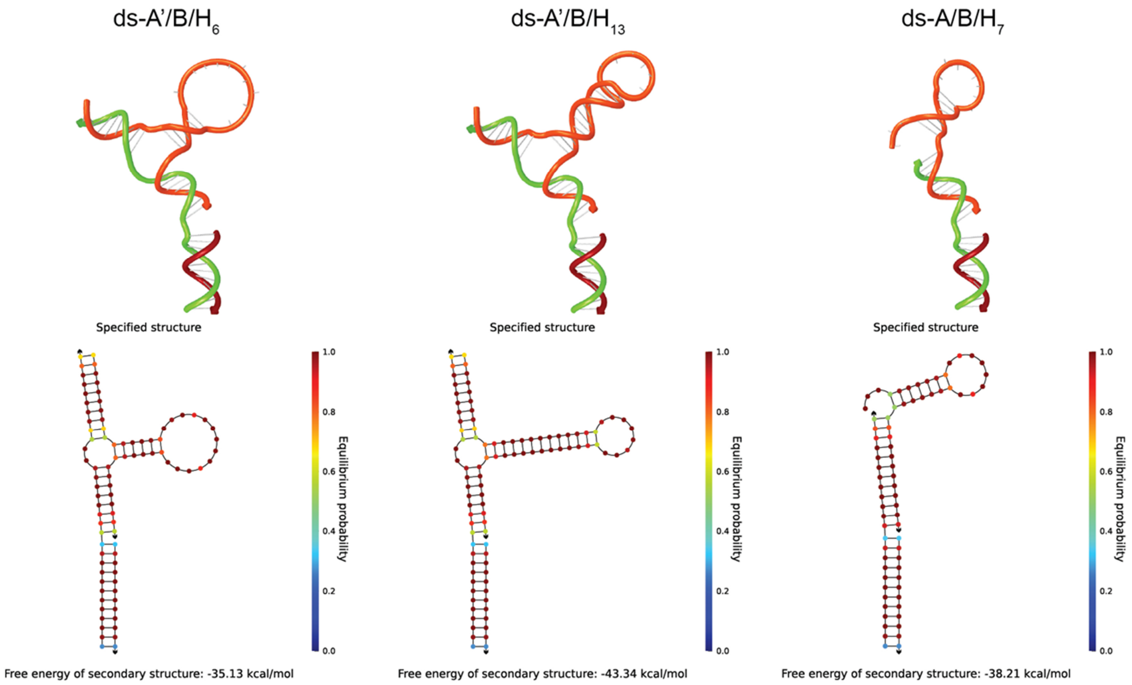

Figure S3. Predicted secondary structures formed by A or A' (long strand), B (short strand) and H (hairpin) using NUPACK analysis algorithms, with associated depiction of equilibrium base-pairing probabilities.[1,2]

Table S2. Properties of the specified structures and sequences.

	Free energy[a] (kcal/mol)	Probability[b]	Ensemble defect[c] (nt)	Normalized ensemble defect[d] (%)	Length (nt)
H_6	-35.1	0.007	8.1	9.3	87
H_{13}	-43.3	0.005	8.7	9.1	96
H_7	-38.2	0.015	6.5	8.8	73

[a]Free energy of the specified secondary structure.
[b]The probability for the formation of this structure.
[c]The average number of nucleotides that are incorrectly paired at equilibrium relative to the specified secondary structure, evaluated over the Boltzmann-weighted ensemble of secondary structures (0 is best, N is worst, for a strand with N bases).
[d]The average percentage of nucleotides that are incorrectly of paired at equilibrium relative to the specified secondary structure (0% is best, 100% is worst).

Subsequently, we studied the hybridization and displacement of the hairpin in solution, with DNA strands not tethered to liposomes. We characterized the formation and disruption of DNA duplexes using native PAGE analysis (Figure S4b). When using dsDNA functionalized with a terminal cholesterol group, we observed that there was no retardation of dsDNA on the gel image (data not shown). This was attributed to the formation of micelles, preventing the migration of dsDNA into the pores of the gel due to their relatively large size.[4] Therefore, we performed the following experiments with DNA without cholesterol functionalization. Equal molar ratio of A (or A') and B were mixed and hybridized, and the hairpin H was also individually formed. Then, equal molar ratio of ds-A/B and H_7, and ds-A'/B and H_6 or H_{13} were mixed and hybridized. Finally, the resulting DNA constructs in solution were diluted, and 5 times molar excess of target miR-29a were added and left to react for 1 h. Each stage was characterized using PAGE (Figure S4b). In the first step, we confirmed that ds-A'/B and ds-A/B were formed successfully (lanes 4 and 5). H_6 was not well hybridized with ds-A'/B (lane 9), but formed a duplex with the target (T) (lane 13). A fraction of H_{13} was hybridized with ds-A'/B (lane 10), but the displacement by T did not occur (lane 14). Only H_7 showed both efficient hybridization with ds-A/B (lane 11) and displacement by T (lane 15), as further evidenced by the regeneration of the band corresponding to ds-A/B. Therefore, we chose H_7 as the hairpin structure for the liposome fusion assay.

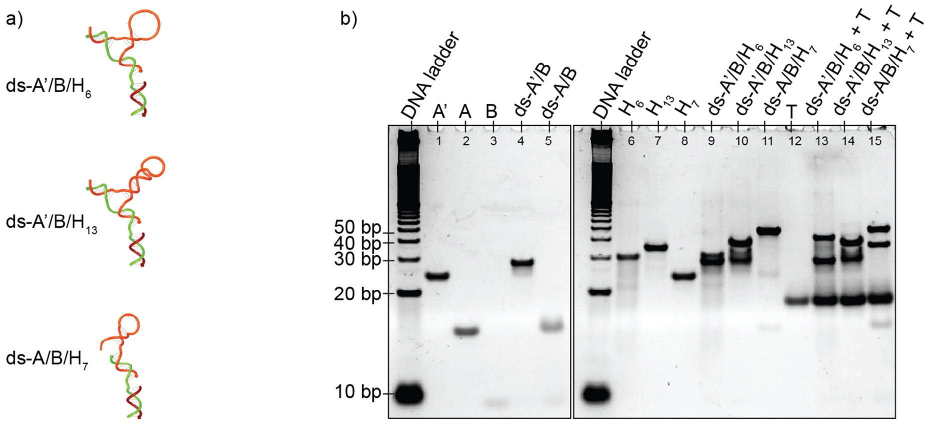

Figure S4. a) Predicted secondary structures formed by A or A' (long ssDNA, green), B (short ssDNA, red) and H (hairpin, orange) using NUPACK analysis algorithms.[1] b) 15 % native PAGE analysis of hairpin hybridization with dsDNA and displacement by target miR-29a (T) of three hairpin designs: H_6, H_{13} and H_7.

S6. Evaluation of the sensitivity and specificity for miR-29a detection

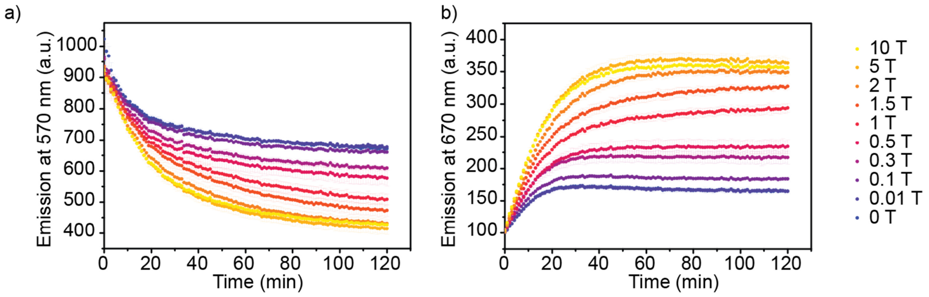

Figure S5. Evolution of fluorescence emission at a) 570 nm, and b) 670 nm when exciting at 530 nm, showing the kinetics of fusion in presence of different quantities of miR-29a (where 1 T corresponds to 2.1×10^{-7} M) (n=3). Error bars represent standard deviation.

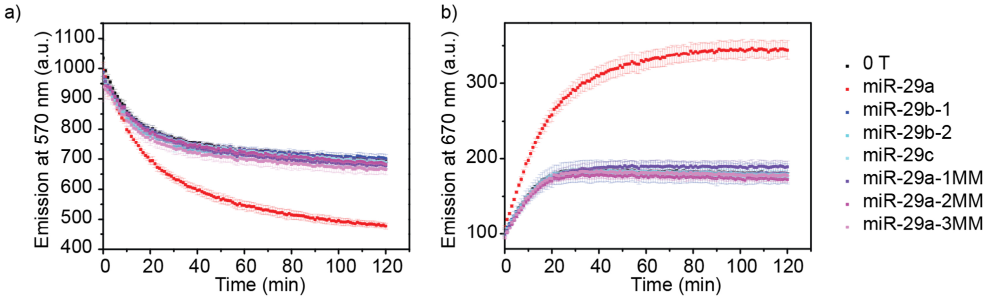

Figure S6. Evolution of fluorescence emission at a) 570 nm, and b) 670 nm when exciting at 530 nm, showing the kinetics of fusion in presence of various miRNA sequences added to DiD liposomes at a concentration of 2.1×10^{-7} M (n=3). See Table S1 for detail of the sequences. Error bars represent standard deviation.

S7. Fluorescence correlation microscopy analysis

The 2-color fluorescence microscopy setup comprises a regular fluorescence microscope equipped with a beam splitter, and relies on the direct excitation of DiI-labeled liposomes by a laser source (488 nm) via an optical fiber inserted in the sample chamber (Figure S7a). As target miRNA displaces hairpin DNA, FRETing fusion complexes are formed. Using a beam splitter and a suitable filter set (Figure S7a), it is possible to separate the emitted light originating from DiI liposomes (centered around 570 nm; denoted as "green channel" in the following) and fused DiI and DiD liposome complexes (centered around 670 nm; "red channel"). The separated channels are then projected onto different parts of the camera sensor, allowing simultaneous imaging of the same field of view using the different emission filters, such that fused liposome complexes exhibiting FRET appear in the red channel, whereas non-fused DiI liposomes only appear in the green channel (Figure S7b, as indicated by the colored boxes).

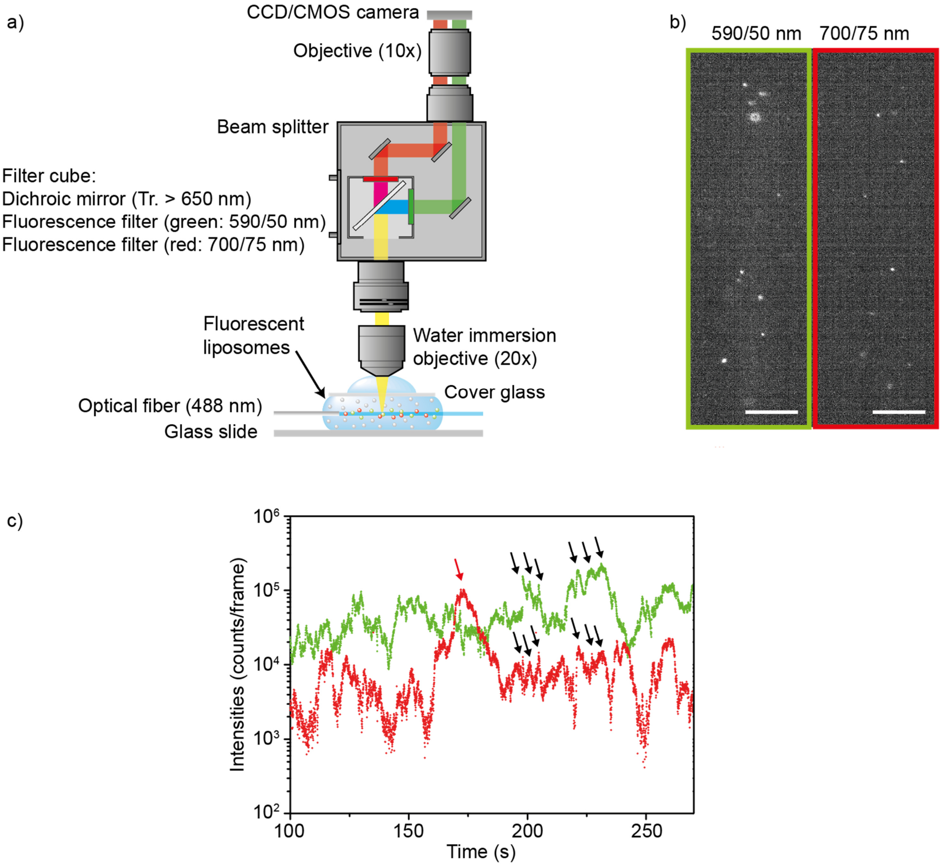

Figure S7. a) Schematic (not to scale) of the 2-color fluorescence microscopy setup. Liposomes in suspension in the sample chamber are excited using a laser source at 488 nm via an optical fiber. A filter cube and beam splitter setup allows the separation of the emitted light into two distinct channels. b) Micrograph snapshot illustrating how the field of view of the camera is split in two. The left side is referred to as the green channel (590/50 nm) and the right as the red channel (700/75 nm) (Scale bars: 25 µm). c) Fluctuations in the intensities $I_g(t)$ and $I_r(t)$ (integrated over the entire green and red channel, respectively) are caused by non-FRETing DiI (black arrows) and fused FRETing liposomes entering and leaving the field of view (red arrow).

The 2-color fluorescence microscopy setup (Figure S7) employed in this study was initially designed to offer the possibility of determining the fluorescence intensity of single liposomes simultaneously at two distinct wavelength intervals, thereby allowing (in theory) to extract FRET ratios of single liposomes that are suspended in bulk solution. This concept was implemented by splitting the field of view (FOV) into two channels using a dichroic mirror and an appropriate set of fluorescence filters, followed by using distinct fluorescence filter sets for each channel, and by identifying the position of a particular liposome in both channels (co-localization) based on the criteria that the random liposome movement must be identical in both channels, indicated by a high correlation of the observed movements. This procedure indeed allowed to extract FRET ratios for the liposomes used in this study, but the extracted FRET ratio distributions were broader than expected and did not permit us to obtain any dose-response curve as in Figure 2b (main text).

Further analysis of this problem showed that the FRET ratio distributions became strongly broadened due to the random movement of the suspended liposomes. This causes liposomes to randomly enter and exit the FOV and a notable fraction of liposomes (those which do not cross the focal plane before leaving the FOV) to be clearly detectable in only one of the two channels, introducing large errors in the extracted FRET ratios. Hence, the random movement, which is used to co-localize liposomes in both channels, also impedes reliable intensity extraction, a shortcoming that has been recently addressed by confining the random liposome movement within a plane.[5]

Nevertheless, the feature of liposomes entering and exiting the FOV is conceptually similar to the method fluorescence correlation spectroscopy (FCS), in which single, dye-labeled objects enter and exit a defined optical readout volume (usually the diffraction-limited spot of a confocal microscope) causing fluctuations in the recorded fluorescence intensity $I(t)$, which are proportional to the temporal fluctuations in $N(t)$, the number of labeled objects within the readout volume.[6–8] If only one species is investigated using such a setup, it can be shown that the time average of $N(t)$,

$$N = \langle N(t) \rangle, \tag{S1}$$

can be extracted from the autocorrelation function $G(\tau)$ of $I(t)$

$$G(\tau) = \frac{\langle I(t) \cdot I(t+\tau) \rangle}{\langle I(t) \rangle^2} \tag{S2}$$

based on the identification

$$G(\tau) = 1 + \frac{1}{N} \cdot \frac{1}{1+\tau/\tau_\mathrm{D}} \cdot \frac{1}{\sqrt{1+z^2\tau/\tau_\mathrm{D}}} \tag{S3}$$

with τ denoting the lag time (used in the calculation of the autocorrelation function), τ_D the object's mean residence time within the confocal readout volume, and z the ratio of radial to axial dimensions of the confocal readout volume.[9] Note that Eq. S3 includes only fluctuations caused by temporal changes in $N(\tau)$, which is the only source of fluctuations that can be resolved using the 2-color fluorescence microscopy setup (acquisition rate ~ 30 Hz). FCS implementations achieve acquisition rates on the order of 10 MHz, making it necessary to include additional contributions in Eq. S3 like triplet dynamics. Nevertheless, the average number of objects within the observation volume, N, can be extracted from Eq. S2 and S3 in the limit of $\tau \rightarrow 0$:

$$N = 1/g(0) \text{ with } g(\tau) = G(\tau) - 1. \tag{S4}$$

Eq. S4 reflects the rare situation that a signal (here the magnitude of the autocorrelation function) becomes enhanced with a decreasing object concentration, which is a consequence of the fact that entering and exiting of a small number of objects in the readout volume creates larger fluctuations in $I(t)$ (with respect to the time average value) than a larger number. Hence, fluctuation-based

approaches typically work best for small N, an advantage that has to be paid by increased observation times to arrive at feasible signal-to-noise ratios.

A direct application of this concept to the liposomes introduced in the main text failed, however, since even in absence of any target DNA liposomes were detectable in the red DiD channel. Most of these liposomes showed weak intensity in the red and strong intensity in the green channel, suggesting a bleed-through mechanism to be responsible for this observation, either caused by non-specific (partial) fusion of DiD and DiI liposomes (inducing small FRETing) or lipid transfer between DiD and DiI liposomes. Indications for this bleed-through mechanism can be found in Figure S7c, showing correlated fluctuations (black arrows) in $I_g(t)$ and $I_r(t)$, *i.e.*, the intensities of the green and red channel, respectively.

As the number concentration of DiI liposomes in our experiments was usually larger than the number concentration of fused FRETing liposomes, a direct application of Eqs S2 - S4 to $I_r(t)$ gives a N value that will be dominated by DiI liposomes. However, taking the bleed-through into account it is possible to express $I_r(t)$ by

$$I_r(t) = \gamma \cdot I_g(t) + \Gamma \cdot N_{FRET}(t) \qquad \text{(S5)}$$

with γ denoting the average bleed-through factor between both channels and $N_{FRET}(t)$ and Γ the number and brightness of FRETing liposomes in the FOV, respectively. This can be further transformed into

$$\frac{I_r(t)}{I_g(t)} = \gamma + \Gamma \cdot \frac{N_{FRET}(t)}{I_g(t)} . \qquad \text{(S6)}$$

Under the assumption that the number concentration of DiI liposomes is larger than the number concentration of fused FRETing liposomes, which is fulfilled in our experiments, the fluctuations in $I_g(t)$ (relative to its average value) will be smaller than the corresponding fluctuations in N_{FRET}, making the autocorrelation function of the ratio

$$R(t) = I_r(t) / I_g(t) - \gamma \qquad \text{(S7)}$$

strongly dependent on N_{FRET}:

$$\lim_{\tau \to 0} \frac{< R(t) \cdot R(t+\tau) >}{< R(t) >^2} \propto \frac{1}{N_{FRET}} . \qquad \text{(S8)}$$

This is illustrated in Figure S8 showing the autocorrelation functions of $I_g(t)$ and $I_r(t)$ for miR-29a concentrations as indicated. Due to bleed-through and the larger number concentration of DiI liposomes with respect to fused FRETing liposomes, the $g(0)$ values are mainly sensitive to the number of DiI liposomes within the field of view, indicated by non-systematic fluctuations of the plateau of the autocorrelation function observed at short lag times. Analyzing instead the ratio of $I_r(t)$ and $I_g(t)$, as introduced by Eq. S7, solves this problem. The average bleed-through factor γ was determined from the distribution of the ratio $I_g(t)$ and $I_r(t)$, typically showing a pronounced peak at 0.065 followed by a broad tail distribution in Figure S8c, motivating to set γ = 0.065 for all measurements shown in Figure S8s. The corresponding autocorrelation function of the intensity ratio $R(t)$ clearly shows a strong dependence of miR-29a concentration on the $g(0)$ values (indicated by a systematic decrease of the autocorrelation function plateau at short lag times for increasing DNA concentration) (Figure S8d).

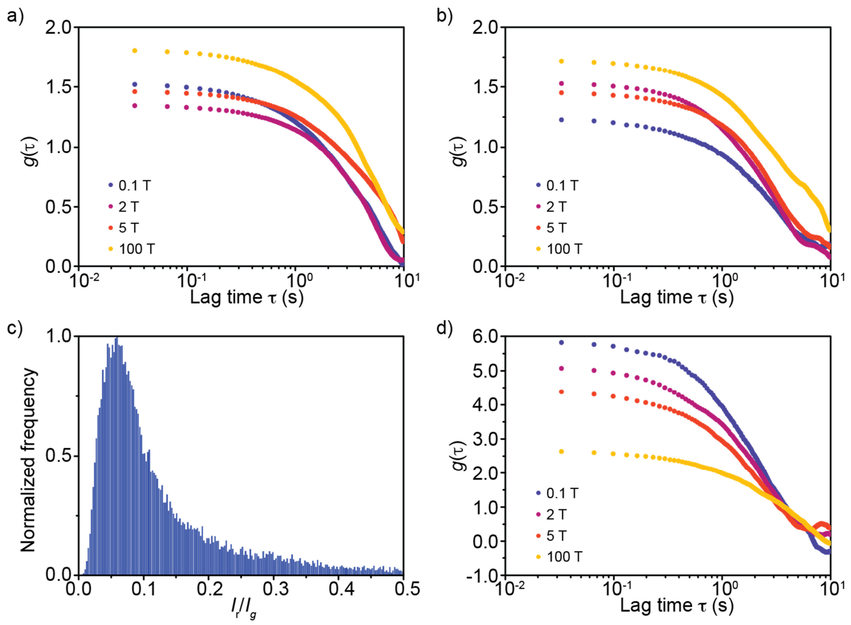

Figure S8. Autocorrelation functions of a) $I_g(t)$, b) $I_r(t)$, and d) $I_r(t)/I_g(t) - \gamma$, with γ = 0.065 as determined from the peak position in c) showing the distribution of the ratio $I_r(t)/I_g(t)$. The intensity traces were recorded from solutions containing 10 pM DiI and DiD liposomes mixed with the indicated amount of miR-29a (where 1 T corresponds to 7.2×10^{-10} M) followed by further dilution to a liposome concentration of 1 pM. Shown are autocorrelation functions for one sample set covering 5 measurements.

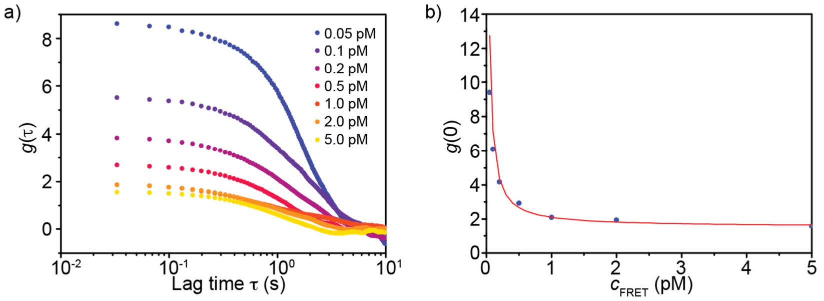

Figure S9. a) Autocorrelation functions of the intensity ratio $I_r(t)/I_g(t)$ for solutions containing only FRETing liposomes (with bulk concentrations c_{FRET} as indicated). This control clearly shows that the $g(0)$ value decreases with increasing concentration of FRETing liposomes, as expected by Eq. S8, although saturation effects are observed for c_{FRET} > 1 pM. b) Extracted $g(0)$ values (open circles) and fit (red line) using the relation $g(0)$ = 0.56 pM/c_{FRET} + 1.55, allowing the bulk concentration of FRETing vesicles to be determined based on the measured $g(0)$ values.

In order to quantitatively extract c_{FRET} from $g(0)$, we performed the same data analysis on additional measurements involving solutions containing only FRETing liposomes of known concentration c_{FRET}. These liposomes were composed of DOPC/DOPE/Cholesterol (50:25:25 mass ratio) and contained 1 mol % of DiI and 1 mol % of DiD. Dry lipid films (5 mg/mL) were hydrated in TE buffer (10 mM Tris, 1 mM EDTA, pH 8.0) containing 150 mM NaCl at 37 °C for 30 min and liposomes were formed by extrusion using the Avanti Lipids Mini-Extruder with 100 nm polycarbonate membranes (Whatman). The liposomes were diluted to appropriate concentration in TE buffer (10 mM Tris, 1 mM EDTA, pH 7.4) containing 150 mM NaCl, before measuring in the 2-color fluorescence correlation microscope (Figure S9), using the same setup as described in the main text.

Based on Eq. S8, it is expected that the $g(0)$ value of the autocorrelation function decreases with increasing N_{FRET}, a trend which is qualitatively observed in Figure S9a. On a quantitative basis, however, deviations from Eq. S8 are observed (Figure S9b), which are attributed to saturation effects in the recorded fluctuations (for c_{FRET} > 1 pM), a feature that is frequently observed in FCS measurements performed at too high analyte concentrations. Nevertheless, these measurements allowed to calibrate the relationship between concentration of FRETing vesicle and $g(0)$ finally yielding

$c_{\mathrm{FRET}} = \dfrac{0.56\ \mathrm{pM}}{g(0) - 1.55}$, allowing to translate the $g(0)$ value of the autocorrelation function into concentrations of FRETing vesicles (Figure 4c in the main text). A fit to all data points (Figure 4c, solid line) finally led to the dose-response curve,

$$c_{\mathrm{FRET}}(\mathrm{pM}) = 0.18 + \frac{0.22}{1 + \dfrac{1.31}{[\mathrm{Target}]}} \tag{S9}$$

with [Target] in nM.

S8. Calculation of the LOD for the 2-color fluorescence microscopy setup

The LOD was calculated from the standard deviation, average value, and fitted dose-response curve (Eq. S9) shown in Fig. 4c in the main text. The blank measurement (absence of target DNA) had an average concentrations of FRETing vesicles of 0.181 pM and a standard deviation of 0.034 pM, which were calculated from 3 independent measurement series. Using the usual definition and the dose-response curve of Eq. S9 leads to a LOD of 1.2 nM for the 2C FCS setup.

References

[1]. J. N. Zadeh, C. D. Steenberg, J. S. Bois, B. R. Wolfe, M. B. Pierce, A. R. Khan, R. M. Dirks, N. A. Pierce. *J. Comput. Chem.*, **2011**, *32*, 170–173.

[2]. NUPACK Nucleic Acid Package http://www.nupack.org (accessed Jan 8, 2016).

[3]. G. Stengel, L. Simonsson, R. A. Campbell, F. Höök. *J. Phys. Chem. B*, **2008**, *112*, 8264–8274.

[4]. A. Johnson-Buck, S. Jiang, H. Yan, N. G. Walter. *ACS Nano*, **2014**, *8*, 5641–5649.

[5]. S. Block, B. J. Fast, A. Lundgren, V. P. Zhdanov, F. Höök. *Nat. Commun.*, **2016**, *7*, 12956–12963.

[6]. E. L. Elson, D. Magde. *Biopolymers*, **1974**, *13*, 1–27.

[7]. D. Magde, E. L. Elson, W. W. Webb. *Biopolymers*, **1974**, *13*, 29–61.

[8]. R. Rigler, Ü. Mets, J. Widengren, P. Kask. *Eur. Biophys. J.*, **1993**, *22*, 169–175.

[9]. J. Langowski. *Methods Cell Biol.*, **2008**, *85*, 471–484.
